# Nationwide Q Fever Hospitalizations in Italy, 2014–2023: Evidence of Underrecognition and a Sardinian Hotspot

**DOI:** 10.1093/ofid/ofag667

**Published:** 2026-10-21

**Authors:** Daniel Cifo, David González-Barrio, Alberto Mateo-Urdiales, Patrizio Pezzotti, Alessandra Ciervo, Angela Fanelli, Alda Natale, Diana Gómez-Barroso, Rosa M Estévez-Reboredo

**Affiliations:** Primary Care Research Unit, Madrid Health Service (SERMAS), Madrid, Spain; Biomedical Sciences and Public Health Doctorate Program, UNED – ENS Mixed Research Institute (IMIENS), Madrid, Spain; National Centre of Microbiology (ISCIII-CNM), Carlos III Health Institute, Madrid, Spain; Consortium for Biochemical Research in Infectious Diseases (CIBERINFEC), Madrid, Spain; Dipartimento Malattie Infettive, Istituto Superiore di Sanità, Rome, Italy; Dipartimento Malattie Infettive, Istituto Superiore di Sanità, Rome, Italy; Dipartimento Malattie Infettive, Istituto Superiore di Sanità, Rome, Italy; European Commission, Joint Research Centre (JRC), Seville, Spain; Facultad de Veterinaria, Universidad Alfonso X el Sabio (UAX), Madrid, Spain; Istituto Zooprofilattico Sperimentale delle Venezie, Legnaro, Italy; National Centre of Epidemiology (ISCIII-CNE), Carlos III Health Institute, Madrid, Spain; Consortium for Biochemical Research in Epidemiology and Public Health (CIBERESP), Madrid, Spain; National Centre of Epidemiology (ISCIII-CNE), Carlos III Health Institute, Madrid, Spain; Consortium for Biochemical Research in Epidemiology and Public Health (CIBERESP), Madrid, Spain

**Keywords:** *Coxiella burnetii*, hospitalization rates, One Health, Q fever, Sardinia

## Abstract

**Background:**

Q fever is a zoonotic disease caused by *Coxiella burnetii.* In Italy, serologic evidence shows exposure in humans and livestock, but surveillance data are sparse, and underreporting is likely. We aimed to characterize Q fever-related hospitalizations in Italy during 2014–2023 and examine environmental risk correlates.

**Method:**

We conducted a nationwide retrospective study using the Italian National Hospital Discharge Database. Hospitalizations with ICD-9-CM code 083.0 as primary or secondary diagnosis were included. We calculated hospitalization rates by age, sex, and region and described clinical characteristics. We linked hospitalizations with climatic, livestock, and land-use data; for Sardinia, we fitted Bayesian spatial regression models including bovine, ovine, and caprine density, temperature, precipitation, and land use.

**Results:**

We identified 428 hospitalizations (67% male), corresponding to a rate of 0.07 per 100 000 person-years. Rates were highest in children and older adults. The overall case fatality ratio was 2.4%. The most frequent Q fever-related concomitant diagnosis was pneumonia. Sardinia had the highest case count and adjusted hospitalization rate, 7-fold the national rate (0.55 vs 0.07). Spatial models for Sardinia showed hotspots in the north of the island and a 12% increase in spatial hospitalization risk per 10 bovine heads/km^2^ (adjusted relative risk 1.12, 95% credible interval 1.03–1.24).

**Conclusions:**

Q fever causes an underrecognized burden of hospitalizations in Italy, with marked regional heterogeneity and a Sardinian hotspot associated with high cattle density areas. Clinical awareness, surveillance, and One Health interventions are warranted in high-risk areas.

## Teaser

Nationwide hospital discharge data show Q fever causes an underrecognized burden in Italy, with highest rates in Piedmont, Lombardy, Lazio, and especially Sardinia, where spatial models link increased hospitalization risk to cattle density and other environmental factors.

Q fever is a zoonosis caused by *Coxiella burnetii*, a highly resilient intracellular pathogen. The primary reservoirs for human infection are goats, sheep, and cattle [1], although *C. burnetii* has also been identified in many domestic and wild species, including at least 7 tick species [2]. Wildlife and ticks are thought to contribute to maintenance of the pathogen's natural cycle [3]. However, the direct role of ticks as efficient vectors remains controversial [4].

Humans are infected via aerosol inhalation [1]. The highest risk arises from exposure to birthing products and abortion material from infected ruminants, but infections can occur up to 20 km from the source because of the organism's environmental persistence and windborne transport [5]. These features suggest that environmental factors such as land use, vegetation, and climate may influence transmission [6]. Foodborne, tickborne, and vertical transmission are possible but much less common. Human-to-human transmission is very uncommon and mainly iatrogenic [1]. Given its environmental stability, very low infectious dose, and ease of airborne dissemination, *C. burnetii* is classified as a Category B potential bioterrorism agent by the US Centers for Disease Control and Prevention and the European Medicines Agency [7, 8].

Acute Q fever is symptomatic in approximately 40% of infected people, often presenting as a nonspecific flu-like illness, likely contributing to underreporting. Retrospective diagnosis of mild forms contributes to discrepancies between seroprevalence estimates and notification rates, especially during outbreaks. Classical acute manifestations, such as atypical pneumonia, febrile hepatitis, and neurologic involvement, are less frequent [9]. An estimated 1%–5% of patients develop chronic/persistent Q fever, most commonly manifesting as endocarditis or vascular infection, with a case fatality rate of approximately 15% [10]. Because routine isolation of *C. burnetii* is rarely feasible, diagnosis relies on specific direct polymerase chain reaction (PCR) assays and indirect serologic assays [1].

The largest recorded outbreak of Q fever occurred in the Netherlands during 2007–2010, with over 4000 reported cases and an estimated 40 000 infections [11]. In Italy, data from the European Centre for Disease Control and Prevention (ECDC) indicate that the annual number of reported human Q fever cases has remained below 10 in recent years [12]. This low notification rate contrasts with the country's historical experience with the disease, including large outbreaks among troops during World War II [13] and more recent events such as a cluster in September 2021 involving 14 tourists at a holiday farm in Bolzano, northern Italy [14].

Seroprevalence studies further support substantial circulation of *C. burnetii* in both humans and livestock. A meta-analysis of serosurveys among people in occupational risk groups in Italy estimated a pooled seroprevalence of 44% [15]. A recent study showed a prevalence ratio of 8.5 for occupational risk groups compared to the general population [16]. Animal surveys have reported 13% seroprevalence in cattle from Apulia, Basilicata, Campania, and Sicily [17]; 38% in sheep from Lazio [18]; and 28% in goats from Piedmont [19]. At a broader scale, Q fever is considered endemic in the Mediterranean, including Spain, France, North Africa, and the Eastern Mediterranean region [20–23].

Against this background of scarce and fragmented serologic and notification data in Italy and given the potential of hospital discharge databases to complement traditional surveillance, we aimed to (1) characterize Q fever-related hospitalizations in Italy during 2014–2023, (2) describe regional and temporal patterns, and (3) examine the role of environmental and livestock-related determinants.

## MATERIALS AND METHODS

### Study Population, Geographical and Temporal Scope

We conducted a retrospective study of all Q fever-related hospitalizations recorded in the National Hospital Discharge Database (NHDD) from 1 January 2014 through 31 December 2023. The study area included the Italian Peninsula, Sicily, and Sardinia: 7906 municipalities across 19 regions and the autonomous provinces of Trento and Bolzano, with a total population of approximately 58.9 million.

### Data Collection

#### Hospitalization Data

We obtained 2014–2023 hospitalization data from the NHDD, maintained by the Italian Ministry of Health. The NHDD contains detailed sociodemographic and clinical information on public and private hospital discharges. Personal identifiers are encrypted, and each patient is assigned a unique code.

Each record includes 1 primary discharge diagnosis and up to 5 secondary diagnoses, coded according to the International Classification of Diseases-Clinical Modification, 9th edition (ICD-CM-9). Patients were included if they had ICD-CM-9 code 083.0 (Q fever) as either primary or secondary diagnosis. We retrieved data on sex, age, birthplace, residence and hospital location (region, municipality, and province), length of stay, discharge type, and concurrent diagnosis codes as an exploratory approach to provide clinical context. To avoid double-counting, we retained only the first hospitalization in chronological order for each patient. Only the first hospitalization per patient was retained. Supplementary Table 1 shows grouping of ICD-9 diagnosis codes for concurrent diagnoses.

#### Climate Data

We obtained mean temperature (°C) and precipitation (mm) data from 1422 Italian climate stations, provided by the Italian Meteorological Agency (ItaliaMeteo). For analytical purposes, each municipality was assigned values from the nearest station within 30 km, considering potential windborne dispersion of *C. burnetii*.

#### Livestock Data

We retrieved livestock census data from the Italian National Institute of Statistics (ISTAT). For each municipality, the dataset included mean annual cattle, sheep, and goat heads. Based on previous studies [6], we calculated livestock density (heads/km^2^) by dividing the total number of animals by the municipality land area.

#### Land-Use Data

We obtained land-use data from the Corine Land Cover 2018 geodatabase, provided by the Copernicus EU Programme. The original dataset includes 47 land-use classes, which we aggregated according to database specifications. We selected the 4 most frequent categories: shrub and herbaceous vegetation, arable land, heterogeneous agricultural areas (eg, agricultural areas mixed with natural vegetation and agroforestry areas), and forests. For each municipality, we calculated the percentage of total area occupied by each category. Supplementary Table 2 provides a breakdown of land-use area.

### Descriptive Analysis

We calculated hospitalization rates (HRs) for Q fever per 100 000 person-years for the overall study period. We estimated crude, age-specific (10-year age groups), and sex-specific HRs with 95% confidence intervals (CIs) at national and regional levels using ISTAT population figures. Annual and monthly HRs were also calculated to assess temporal patterns. Continuous variables were summarized as mean and standard deviation (SD) (or median and interquartile range [IQR] for non-normally distributed variables), and categorical variables as counts and percentages.

### Spatial Analysis

The spatial analysis included 2 components: (1) initial spatial smoothing and (2) a final spatial regression model incorporating environmental and livestock-related covariates. Based on descriptive results showing a marked predominance of hospitalizations in Sardinia, spatial analysis was restricted to Sardinia.

The unit of analysis was the municipality of residence. For 8 of 60 hospitalization cases identified in Sardinia (13%), information on the municipality of residence was missing; for these, we used the municipality of birth.

#### Spatial Smoothing

We calculated municipal-level standardized hospitalization ratios (SHRs) by indirect age standardization, using the 2013 European standard population to derive the expected number of cases. To smooth SHR values, accounting for spatial correlation, we fitted a Besag-York-Mollié (BYM) conditional autoregressive model using Integrated Nested Laplace Approximation (INLA) Bayesian estimation to obtain crude spatial relative risks (cRR) for each municipality. Expected cases were used as an offset. SHR, cRR, and their corresponding credible intervals (CrIs) were displayed in choropleth maps.

#### Spatial Regression

To estimate adjusted spatial relative risks (aRR) and assess associations with environmental and livestock factors, we fitted a BYM spatial regression model. Selected covariates included mean temperature (°C), mean precipitation (mm), bovine, ovine, and caprine density (heads/km^2^), land use dedicated to shrub and herbaceous vegetation (%), arable land (%), heterogeneous agricultural areas (%), and forest (%). Covariates were selected based on previous studies in other settings [6, 24] and data availability. The BYM regression produced municipal aRR values and CrIs, displayed in choropleth maps. Additionally, we reported exponentiated regression coefficients for covariates as relative risks with 95% CrIs.

### Software

We conducted epidemiologic analyses (HRs, summary statistics, and regression models) using R version 4.3.0 (R Foundation for Statistical Computing) [25]. Spatial models were fitted with the R-INLA version 24.03.29 [26], and we produced choropleth maps using QGIS version 3.24.3 [27].

### Ethical Considerations

Ethics approval was not required for this study because the analysis was based on routinely collected, anonymized hospital discharge data without identifiable patient information.

## RESULTS

### Descriptive Analysis

#### Overview and Hospitalization Characteristics

From 2014 through 2023, 428 Q fever-related hospitalizations were recorded in Italy, corresponding to an overall HR of 0.07 (95% CI 0.07–0.08). Males accounted for 287 cases (67%) with an HR of 0.10 (95% CI 0.09–0.11), while females represented 141 cases (33%) and an HR of 0.05 (95% CI 0.04–0.06). Age ranged from 0 to 89 years. The highest HRs were observed among adults in the 75–84 age group, with an HR of 0.11 (95% CI 0.08–0.14) and 54 hospitalizations, followed by the 65–74 age group, with an HR of 0.11 (95% CI 0.08–0.13) and 73 cases. Table 1 presents case counts, HRs, and 95% CIs by sex and age group.

**Table 1.** Number of Q Fever-Related Hospitalizations and Hospitalization Rates by Age Group and Sex, Italy, 2014–2023

Age Group (years)	No. of Hospitalizations			Hospitalization Rate^a^, per 100 000 p-y^‡^(CI^b^ 95%)		
	Male	Female	Total	Male	Female	Total
<15	35	30	65	0.09 (0.06–0.13)	0.08 (0.06–0.12)	0.09 (0.07–0.11)
15–24	13	13	26	0.04 (0.02–0.07)	0.05 (0.02–0.08)	0.04 (0.03–0.07)
25–34	21	11	32	0.07 (0.04–0.10)	0.04 (0.02–0.07)	0.05 (0.04–0.07)
35–44	26	8	34	0.07 (0.05–0.11)	0.02 (0.01–0.04)	0.05 (0.03–0.07)
45–54	36	19	55	0.08 (0.05–0.11)	0.04 (0.02–0.06)	0.06 (0.04–0.08)
55–64	49	21	70	0.11 (0.08–0.15)	0.05 (0.03–0.07)	0.08 (0.06–0.10)
65–74	57	16	73	0.17 (0.13–0.23)	0.04 (0.03–0.07)	0.11 (0.08–0.13)
75–84	37	17	54	0.17 (0.12–0.23)	0.06 (0.04–0.10)	0.11 (0.08–0.14)
>84	13	6	19	0.17 (0.09–0.28)	0.04 (0.01–0.09)	0.08 (0.05–0.13)
Total	287	141	428	0.10 (0.09–0.11)	0.05 (0.04–0.06)	0.07 (0.07–0.08)

^a^Rates are for the entire 2014–2023 period.

^b^CI, confidence interval.

^‡^p-y, person-years.

The mean age of hospitalized patients was 49 years (SD 26), and the median length of hospital stay was 10 days (IQR 5–18). The most frequent destination at discharge was discharge at home (382/428, 89%), followed by voluntary discharge (16/428, 3.7%), and transfer to another unit (16/428, 3.7%). Ten deaths were recorded, corresponding to a case fatality ratio of 2.3%. Regarding concurrent diagnosis codes, 58 cases (13.6%) had a pneumonia-related code in addition to Q fever, and 44 (10.3%) had an endocarditis/pericarditis-related code. Table 2 summarizes the characteristics of hospitalized patients.

**Table 2.** Characteristics of Hospitalized Q Fever-Related Cases, Italy, 2014–2023

Diagnosis Type	Total (No. = 428)
Sex, No. (%)	**…**
Male	287 (67%)
Female	141 (33%)
Age, mean (SD^a^)	49 (26)
Stay, days, median (IQR^b^)	10 (5, 18)
Type of discharge, No. (%)	**…**
Home	382 (89%)
Voluntary	16 (3.7%)
Transfer	16 (3.7%)
Death	10 (2.3%)
Social-sanitary center	3 (0.7%)
Hospital at home	1 (0.2%)
Concurrent diagnoses, No. (%)	…
Pneumonia	58 (13.6%)
Endocarditis and pericarditis	44 (10.3%)
Hepatitis	14 (3.3%)
Vascular disease	8 (1.9%)
Meningitis	2 (0.5%)
Chronic pain	1 (0.2%)

^a^SD, standard deviation.

^b^IQR, interquartile range.

#### Regional and Temporal Distribution

Age- and sex-adjusted HRs varied markedly by region. Sardinia had the highest adjusted HR (0.55; 95% CI 0.42–0.71) with 60 hospitalizations (14%). Piedmont and Marche followed with adjusted HRs of 0.27 (95% CI 0.20–0.35; 60 cases) and 0.22 (95% CI 0.11–0.40; 12 cases), respectively. Valle d’Aosta was the only region with no recorded hospitalizations. Table 3 presents case counts, crude HRs, and adjusted HRs by region.

**Table 3.** Number of Q Fever-Related Hospitalizations and Hospitalization Rates by Region, Italy, 2014–2023

Region	No. of Hospitalizations	Crude Hospitalization Rate^a^ (CI^b^ 95%)	Age- and Sex-Adjusted Hospitalization Rate^a^ (CI^b^ 95%)
Sardinia	60	0.38 (0.29–0.49)	0.55 (0.42–0.71)
Piedmont	60	0.14 (0.11–0.18)	0.27 (0.20–0.35)
Veneto	59	0.12 (0.09–0.16)	0.19 (0.14–0.25)
Lombardy	56	0.06 (0.04–0.07)	0.08 (0.06–0.10)
Campania	43	0.08 (0.06–0.10)	0.15 (0.11–0.20)
Lazio	37	0.06 (0.05–0.09)	0.12 (0.09–0.17)
Sicily	26	0.05 (0.04–0.08)	0.11 (0.07–0.16)
Emilia-Romagna	19	0.04 (0.03–0.07)	0.09 (0.06–0.15)
Marche	12	0.08 (0.04–0.14)	0.22 (0.11–0.40)
Calabria	11	0.06 (0.03–0.11)	0.16 (0.08–0.29)
Liguria	8	0.05 (0.02–0.10)	NA
Friuli-Venezia Giulia	7	0.06 (0.02–0.12)	NA
Trento (AP^c^)	6	0.11 (0.04–0.24)	NA
Umbria	6	0.07 (0.03–0.15)	NA
Toscana	5	0.01 (0.00–0.03)	NA
Molise	5	0.17 (0.06–0.40)	NA
Apulia	3	0.01 (0.00–0.02)	NA
Basilicata	2	0.04 (0.00–0.13)	NA
Bolzano/Bozen (AP^c^)	2	0.04 (0.00–0.14)	NA
Abruzzo	1	0.01 (0.00–0.04)	NA
Valle d’Aosta	0	0	0
Total	428	0.07 (0.07–0.08)	0.07 (0.07–0.08)

^a^Rates are calculated per 100 000 person-years for the entire 2014–2023 period. Adjusted rates were not calculated for regions with <10 cases because of statistical unreliability.

^b^CI, confidence interval.

^c^AP, autonomous province.

 The year with the highest number of hospitalizations was 2019 (68 cases), followed by 2018 (51 cases) and 2023 (48 cases). In 2019, 32 (47%) hospitalizations had Q fever coded as the primary diagnosis; the remaining 36 (53%) were secondary diagnoses. No clear long-term upward or downward trend was observed. A peak in mid-2019, largely driven by secondary cases, was followed by a return to previous levels in 2020. A possible seasonal pattern was observed, peaking in spring and summer (Figure 1).

**Figure 1.**
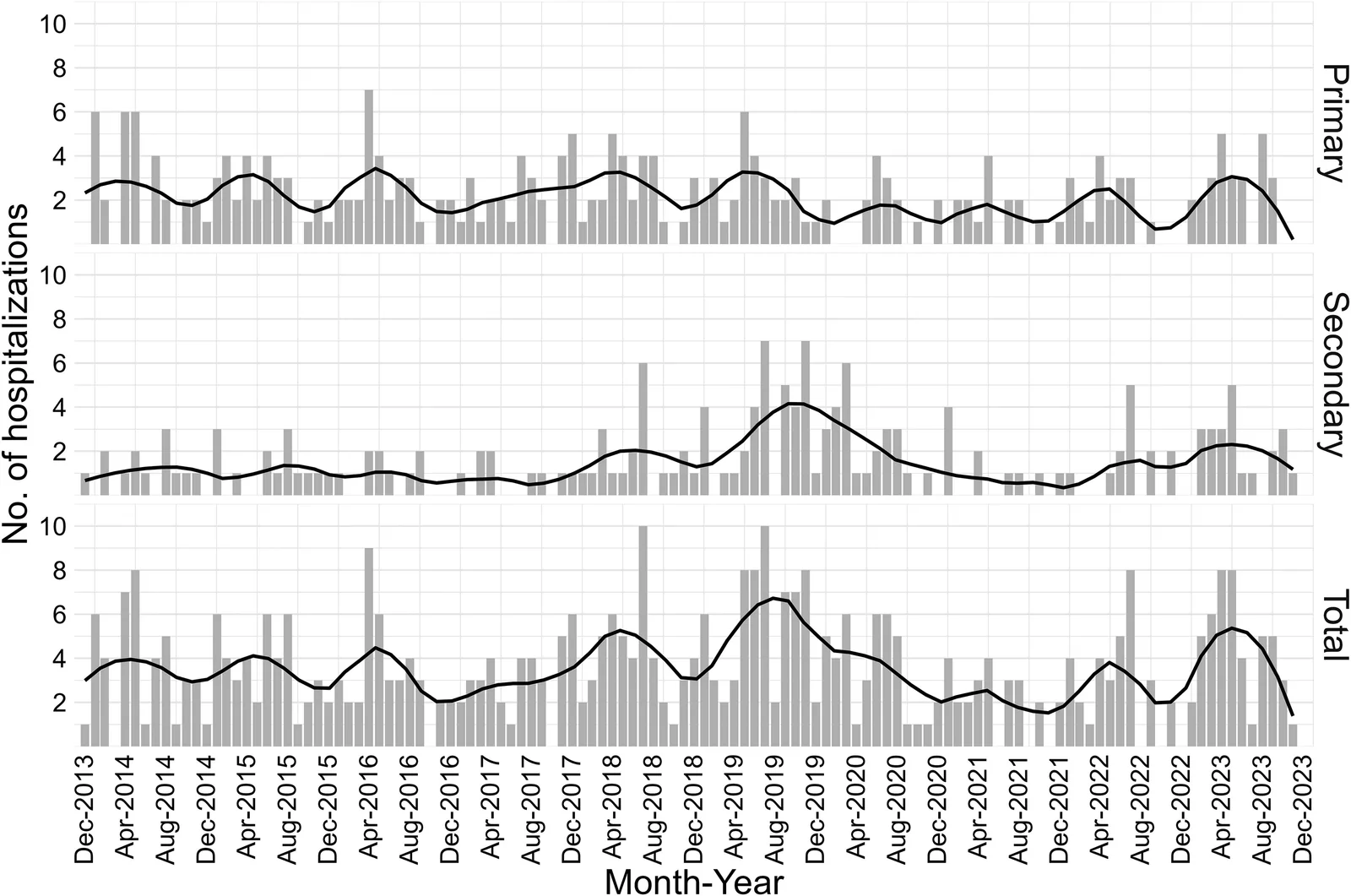
Monthly number of Q fever-related hospitalizations and LOESS (locally estimated scatterplot smoothing) curves, by diagnosis type, Italy, 2014–2023.

### Spatial Analysis

Because case numbers were scarce in most Italian regions, spatial analyses were restricted to Sardinia.

#### Spatial Smoothing

Municipalities with higher SHRs were mainly located in the northern half of the island (Figure 2, left). After spatial smoothing, the cRRs were highest in the northwest around Sassari, with additional hotspots along the eastern coast near Lanusei and Ilbono in the province of Ogliastra (Figure 2, above).

**Figure 2.**
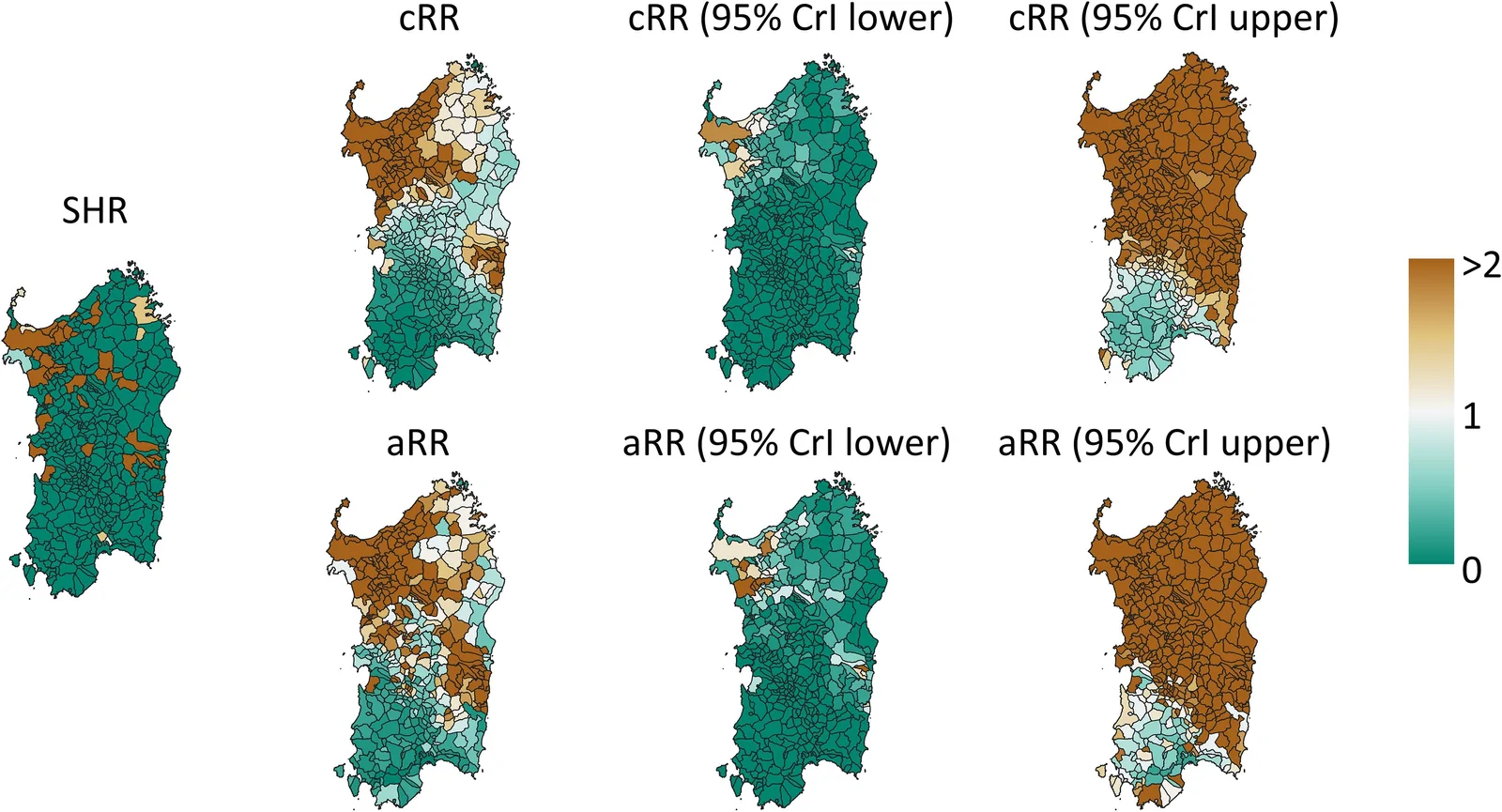
Spatial distribution of Q fever-related hospitalization risk in Sardinia, Italy, 2014–2023. Standardized hospitalization ratio (SHR, left). Crude relative risk (cRR), lower bound of the 95% credible interval (CrI) of cRR and upper bound of the 95% CrI of cRR (above). Adjusted relative risk (aRR), lower bound of the 95% CrI of aRR and upper bound of the 95% CrI of aRR (below). Models adjusted by caprine, bovine, and ovine density (heads/km^2^), mean temperature (°C), mean precipitation (mm), percentage land use dedicated to arable land, shrub and herbaceous vegetation, heterogeneous agricultural areas, and forest.

#### Spatial Regression

In the adjusted spatial regression model, elevated aRRs persisted in these hotspots. Additional high-risk areas were identified around Alghero (northwest) and in the north-central interior, including the municipalities of Anela, Bultei, and Nule (province of Sassari) and Orune (province of Nuoro) (Figure 2 below). Spatial distributions of covariates are available in Supplementary Figure 1.

A 10-heads/km^2^ increase in bovine density was spatially associated with a 12% increase in hospitalization risk (aRR 1.12, 95% CrI 1.03–1.24). The proportion of land use classified as heterogeneous agricultural areas was also positively associated with risk (aRR 1.05, 95% CrI 1.01–1.09). Shrub and herbaceous vegetation (aRR 1.02, 95% CrI 0.99–1.06) and arable land (aRR 1.01, 95% CrI 0.98–1.05) showed small, imprecise positive associations. Mean temperature was negatively, but not significantly, associated with risk (aRR 0.83, 95% CrI 0.66–1.04). Neither ovine density, caprine density, forested land, nor mean precipitation showed clear association with Q fever hospitalization risk. Supplementary Table 3 provides a full overview of the regression results.

## DISCUSSION

Q fever is a paradigmatic One Health challenge: *C. burnetii* has numerous animal reservoirs, marked environmental resistance, airborne transmission, and high human susceptibility [1]. In Italy, scarce European Food Safety Authority (EFSA)-ECDC data on human Q fever likely reflect surveillance limitations rather than true absence of the disease [28]. Because NHDD reporting is mandatory, hospital discharges provide a useful proxy for epidemiology. Our study identifies 3 key findings: sustained Q fever hospitalization burden despite low notifications, marked regional heterogeneity, especially in Sardinia, and ecological associations with cattle density and environmental conditions.

At the patient level, most of the 428 Q fever-related hospitalizations identified occurred in males, with a bimodal age distribution peaking in children (<15 years) and older adults (>65 years). Surveillance systems typically show a similar male excess but an age distribution skewed toward middle-aged adults [29]. Male predominance, also seen for zoonoses such as tularemia, likely reflects sex- or gender-differential occupational and recreational exposures, together with behavioral and healthcare access factors [15, 30]. The difference between hospitalizations and notifications age profiles is consistent with greater disease severity and admission likelihood at age extremes, even if incident infections are concentrated in working-age adults due to occupational risk [1].

Among concomitant diagnosis codes, pneumonia and endocarditis/pericarditis were the most frequent categories compatible with recognized clinical manifestations, whereas hepatitis and other manifestations were less common. This may reflect clinical recognition and coding patterns: atypical pneumonia is more readily identifiable through clinical and radiological findings [31, 32], while Q fever-associated hepatitis often presents as nonspecific fever with liver enzyme abnormalities [9]. The relatively high proportion of endocarditis/pericarditis, the main chronic manifestation, likely reflects the selective nature of hospitalization data, which capture severe or clinically recognized disease rather than the full infection spectrum [33]. However, these results are mainly exploratory and should be considered with caution. The slightly higher case fatality ratio observed here, compared with previous reports [34, 35], may also reflect selection toward more severe hospitalized cases.

Case counts were highest in Sardinia, northern Italy (Piedmont, Veneto, and Lombardy, where much of the national livestock industry is concentrated) and, to a lesser extent, in Campania and Lazio. Because most of these regions are highly populated, higher counts are expected. However, Sardinia clearly stood out in crude and adjusted HRs, with more than double the adjusted HR of Piedmont and more than 7 times the national adjusted HR (Italy HR: 0.07; Sardinia HR: 0.55). A recent island-wide serological study covering the same period estimated 27% seroprevalence in the general population [36], comparable to or higher than selected occupational risk groups elsewhere in Italy (25% in Toscana to 49% in Apulia) [15]. This supports high exposure on the island, although greater local awareness of *C. burnetii* may contribute to detection.

Animal serosurveys indicate widespread *C. burnetii* exposure across Italy [14, 17–19], including regions with few hospitalizations, such as Calabria, Basilicata, and Apulia (fewer than 20 admissions recorded over 10 years). EFSA 2020 data based on real-time PCR (RT-PCR) testing of vaginal swabs and bulk-tank milk showed herd-level positivity of 19% in sheep and 16% in goats and animal-level positivity of 35% in cattle [37]. Thus, despite widespread animal circulation, human disease remains sparse and heterogeneous, probably because acute Q fever is asymptomatic in 60% of infections and because persistent aerosols can disperse widely from point sources [5]. Distinguishing background environmental contamination from conditions that sustain human transmission remains challenging and should be considered when interpreting surveillance and hospitalization data.

Over time, no clear upward or downward trend was observed, suggesting overall stability. However, a possible seasonal pattern occurred, particularly for primary-diagnosis admissions, with spring peaks and autumn declines. Because primary-diagnosis codes likely capture contacts with the healthcare system closer to the time of infection, this seasonality accords with patterns reported elsewhere [21]. Possible mechanisms include spring meteorology favoring aerosolization and transport, the lambing-related increases in *C. burnetii* shedding, and more outdoor or farm-related activities during warmer months [38].

Given the excess risk in Sardinia, municipal-level spatial analyses were restricted to the island. Spatial smoothing showed a north-south gradient and 2 hotspots: the northwestern tip and eastern coast. Sardinia's complex topography may create microclimatic and ecological heterogeneity that concentrates ruminant holdings near population centers, as reported in other settings (eg, Canary Islands, Balearic Islands, Basque Country, Greece). In addition, *C. burnetii* genotypes vary in host range and human pathogenicity and may be spatially structured [39, 40]. Recent human serology from Sardinia showed a spatial pattern mirroring hospitalizations, with similar hotspots and a north-south gradient [36]. This concordance supports the use of admissions as a proxy for underlying burden and suggests a genuine spatial distribution rather than random variation.

Within Sardinia, climatic and livestock-related factors showed distinct associations with hospitalization risk. Temperature showed a negative, nonsignificant association, contrasting with studies linking transmission of temperature-resistant *C. burnetii* to warmer conditions and spring peaks [6, 24]. This may be explained by the use of mean annual temperatures, which can obscure short, high-risk windows (eg, windy spring days), and by Sardinia's heterogeneous terrain where cooler, windier northern regions showed higher risk. These hypotheses are tentative and require further confirmation. Mean precipitation showed no association. Although not all were statistically significant, the positive associations for arable land, shrub or herbaceous vegetation, and heterogeneous agricultural areas may indicate that limited vegetation cover contributes to environmental persistence and airborne spread of *C. burnetii*, as proposed elsewhere [6]. Further studies are needed to confirm this mechanism in Italy.

Regarding livestock, the strongest spatial association was with cattle: a 12% increase in risk per additional 10 heads/km^2^. This was unexpected because cattle are less numerous than sheep island-wide (100 vs 1600 heads/km^2^ on average) [41]. Ovine and caprine density were not associated with risk. These patterns suggest that context-specific interactions among animal management, environment, and circulating *C. burnetii* genotypes, rather than total ruminant numbers alone, shape human risk. Future work characterizing the spatial distribution of human *C. burnetii* genotypes could help clarify these livestock-specific associations.

Our study has several strengths. Utilizing a nationwide, mandatory database (NHDD) with standardized ICD-9 coding over 10 years favored internal consistency. Additionally, triangulation with ecological covariates provides geographical insights that align with independent serological findings. However, the true burden of Q fever in Italy is likely underrecognized. The annual number of Q fever-related hospitalizations registered in the NHDD is fewer than 70, representing probably only a fraction of the actual disease burden.

Hospital records are not designed for epidemiologic case ascertainment. Coding reflects clinical and administrative priorities, and misclassification is possible. Complete characterization would require microbiologic information, symptoms, and exposure history, which were unavailable in the data sources. Hospitalization data also bias the study toward severe cases. Spatially aggregated data are subject to the modifiable areal unit problem and ecological fallacy; patterns are affected by the level of granularity of the data [42]. Some bias toward higher risk in larger population centers is expected, even when using residence as the place of the case. In addition, the use of place of birth in 8 Sardinian cases with missing information may be a source of misclassification. Regarding livestock data, collinearity between bovine density and specific environmental features, such as windy or mountainous areas, may have influenced the results. Furthermore, in Sardinia, sheep transhumance, which involves the seasonal movement of flocks, may obscure spatial associations. Unfortunately, this dynamic was not captured by our model due to a lack of data. Finally, climatic exposures were measured at large temporal scales (annual means) and may obscure short windows of risk; environmental unmeasured confounders, such as dust events and husbandry practices, could also apply.

In conclusion, despite low notification counts, Q fever caused a sustained hospitalization burden in Italy over the past decade. Risk is higher in populous, livestock-dense regions such as Lazio, Piedmont, Lombardy, Veneto, and Campania, particularly in Sardinia. Because risk is shaped by local environmental conditions and practices, responses should be tailored to identified hotspots by reinforcing surveillance, standardizing biosecurity around parturition and manure management, and considering targeted animal vaccination [43, 44].

Implementation within an integrated One Health framework, including interoperable databases and multidisciplinary prevention involving public health, veterinary, and environmental sectors, could better capture the true burden and reduce human risk.

## Supplementary Material

ofag667_Supplementary_Data

## Supplementary Data

Supplementary materials are available at *Open Forum Infectious Diseases* online. Consisting of data provided by the authors to benefit the reader, the posted materials are not copyedited and are the sole responsibility of the authors, so questions or comments should be addressed to the corresponding author.
