# Supplementary material for: Nationwide Q Fever Hospitalizations in Italy, 2014–2023: Evidence of Underrecognition and a Sardinian Hotspot
Source: Open Forum Infect Dis. 2026 Oct 21;13(10):ofag667. doi: 10.1093/ofid/ofag667 (PMC13684407; doi:10.1093/ofid/ofag667)
Supplement: Supplementary file 1 [file ofag667_supplementary_data.docx]

**Supplementary Table 1: Grouping of ICD-9 diagnosis codes related to Q fever**

| Group | ICD-9 code |
| --- | --- |
| Chronic Pain | 338.2 |
| Endocarditis and pericarditis |  |
| Acute and subacute bacterial endocarditis | 421.0 |
| Acute and subacute infective endocarditis in diseases classified elsewhere | 421.1 |
| Acute endocarditis, unspecified | 421.9 |
| Acute pericarditis in diseases classified elsewhere | 420.0 |
| Acute pericarditis, unspecified | 421.9 |
| Heart valve replaced by other means | V43.3 |
| Mitral valve disorders | 424.0 |
| Other acute pericarditis | 420.99 |
| Other septicemia due to gram-negative organisms | 038.49 |
| Other complications due to heart valve prosthesis | 996.71 |
| Unspecified disease of the pericardium | 423.9 |
| Unspecified septicemia | 038.9 |
| Hepatitis |  |
| Hepatitis in other infectious diseases classified elsewhere | 573.2 |
| Hepatitis, unspecified | 573.3 |
| Nonspecific elevation of levels of transaminase or lactic acid dehydrogenase (LDH) | 790.4 |
| Unspecified disorder of the liver | 573.9 |
| Meningitis |  |
| Meningitis due to gram negative bacteria, not elsewhere classified | 320.82 |
| Meningitis in other bacterial diseases classified elsewhere | 320.7 |
| Pneumonia |  |
| Acute and chronic respiratory failure | 518.84 |
| Acute respiratory failure | 518.81 |
| Bacterial pneumonia, unspecified | 482.9 |
| Bronchopneumonia, organism unspecified | 485 |
| Chronic respiratory failure | 518.83 |
| Other specified alveolar and parietoalveolar pneumonopathies | 516.8 |
| Pneumonia due to other specified bacteria | 482.89 |
| Pneumonia due to other specified organism | 483.8 |
| Pneumonia in other infectious diseases classified elsewhere | 484.4 |
| Pneumonia, organism unspecified | 486 |
| Vascular disease |  |
| Blood vessel replaced by other means | V43.4 |
| Infection and inflammatory reaction due to cardiac device, implant, and graft | 996.61 |
| Other anomalies of aorta | 747.29 |
| Personal history of diseases of circulatory system | V12.5 |
| Personal history of surgery to heart and great vessels, presenting hazards to health | V15.1 |
|  |  |

**Supplementary Table 2: Percentage distribution of land use surface, Sardinia, Italy, 2018**

| Land use | % total surface |
| --- | --- |
| Shrub and herbaceous vegetation | 30.2 |
| Sclerophyllous vegetation | 26.9 |
| Natural grasslands | 2.7 |
| Transitional woodland-shrub | 0.4 |
| Moors and heathland | 0.2 |
| Arable land | 22.3 |
| Non-irrigated arable land | 22.0 |
| Rice fields | 0.2 |
| Permanently irrigated land | 0.1 |
| Heterogeneous agricultural areas | 20.4 |
| Land occupied by agriculture with significant areas of natural vegetation | 9.8 |
| Agro-forestry areas | 7.1 |
| Complex cultivation patterns | 3.4 |
| Annual crops associated with permanent crops | 0.1 |
| Forest | 17.3 |
| Broad-leaved forest | 14.3 |
| Coniferous forest | 2.4 |
| Mixed forest | 0.6 |
| Permanent crops | 2.6 |
| Olive groves | 1.8 |
| Fruit trees | 0.4 |
| Vineyards | 0.4 |
| Urban fabric | 1.8 |
| Discontinuous urban fabric | 1.7 |
| Continuous urban fabric | 0.1 |
| Open spaces with little or no vegetation | 1.8 |
| Sparsely vegetated areas | 1.0 |
| Burnt areas | 0.5 |
| Bare rocks | 0.2 |
| Beaches, dunes, sands | 0.1 |
| Pastures | 1.7 |
| Other | <2.0 |
| Data source: Corine Land Cover 2018. Copernicus EU Programme |  |

**Supplementary Table 3: Adjusted relative risks for Q fever-related hospitalization by environmental and livestock covariates, Sardinia, Italy, 2014-2023**

| Variable | aRR* | 95% CrI† |
| --- | --- | --- |
| **Bovine density, per 10 heads/km2** | **1.12** | **1.03, 1.24** |
| Caprine density, per 10 heads/km2 | 1.07 | 0.75, 1.53 |
| Ovine density, per 10 heads/km2 | 1.00 | 0.96, 1.04 |
| Mean temperature, ºC | 0.83 | 0.66, 1.04 |
| Mean precipitation, mm | 2.01 | 0.63, 6.13 |
| Arable land, % land use | 1.01 | 0.98, 1.05 |
| Shrub and herbaceous vegetation, % land use | 1.02 | 0.99, 1.06 |
| **Heterogeneous agricultural areas, % land use** | **1.05** | **1.01, 1.09** |
| Forest, % land use | 0.98 | 0.94, 1.04 |

*aRR, adjusted relative risk calculated from BYM spatial regression model; †CrI, Credible interval.

**Supplementary Figure 1: Spatial distribution of covariates included in the spatial regression model for Q fever-related hospitalization risk in Sardinia, Italy, 2014-2023. Mean temperature (ºC, above left), mean precipitation (mm, above center), main land use category (highest %, above right), bovine density (heads/km^2^, below right), ovine density (heads/km^2^, below center) and caprine density (heads/km^2^, below left).**

Alt text for supplementary figure 1: Six maps of Sardinia by municipality showing spatial covariates used in the Q fever hospitalization risk model for 2014-2023. The top row shows mean annual temperature, mean annual precipitation and the dominant land use category. The bottom row shows bovine density, ovine density and caprine density, each in heads per square kilometer.
